# Chondroma of laryngeal cartilage mimicking thyroid tumor: A case report

**DOI:** 10.1097/MD.0000000000015005

**Published:** 2019-03-15

**Authors:** ZiQiao Tan, Mengwei Yao, Tao Liu, Shaohua Wang, Jing Xu, Yonghong Zhang, Xinxin Yang, Dengdian Ma, Xiaoyu Li

**Affiliations:** a School of Clinical Medicine Jining Medical University; b Departments of Otolaryngology-Head and Neck Surgery; c Departments of Gynecology, The Affiliated Hospital of Jining Medical University, Jining, China.

**Keywords:** cervical mass, chondroma of laryngeal cartilage, misdiagnosis, surgery

## Abstract

**Rationale:**

Cartilaginous tumors of the larynx are rare. This report describes an atypical case with chondroma of laryngeal cartilage presenting as cervical mass, which was misdiagnosed as a thyroid tumor.

**Patient concern:**

A 73-year-old Chinese man with a 1-month history of cervical mass. The neck color Doppler ultrasound and CT of thyroid showed a space-occupying lesion in the upper right pole of the thyroid gland.

**Diagnoses:**

Chondroma of laryngeal cartilage was confirmed at the time of surgery.

**Interventions:**

After relevant examinations, subtotal thyroidectomy and excisional biopsy of the neck mass were performed under general anesthesia. However, the rapid pathology of the tumor (thyroid right lateral lobe) indicated chondroma, so the patient underwent laryngeal chondroma resection and tracheotomy under general anesthesia.

**Outcomes:**

After surgery, given the advanced age of the patient, long surgical duration and poor cardiorespiratory function, the patient suffered sudden cardiac death after the operation.

**Lessons:**

Cartilaginous tumor of the larynx is rare, and approximately 250 cases have been reported till date. It is difficult to diagnose cartilaginous tumors of the larynx in the early stage, and they are easily misdiagnosed. Early diagnosis, radical surgery, and long-term follow-up are key to prolong survival.

## 1 Introduction

Cartilaginous tumors of the head and neck are rare. Primary chondroid tumors of the larynx represent less than 1% of all laryngeal tumors.^[1]^ Cartilaginous tumors of the larynx most commonly involve the cricoid cartilage. Thyroid and arytenoid cartilage chondromas are extremely rare. In this report, we describe a case with thyroid arytenoid cartilage chondroma misdiagnosed as a thyroid tumor.

## 2 Case report

**Figure 1:**
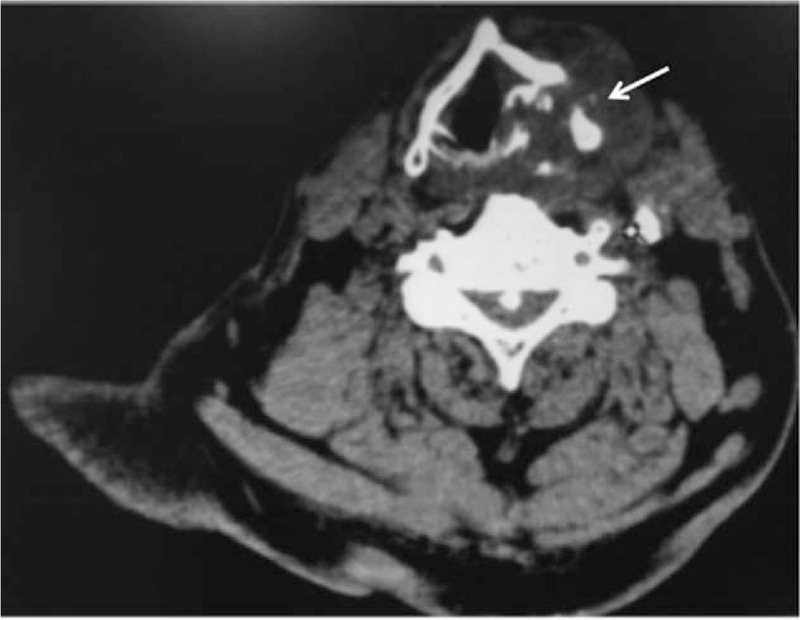
The CT of thyroid showed that the right thyroid lump and multiple calcifications, the thyroid cartilage was compressed and became deformed and reached the right subglottic region. CT = computed tomography.

**Figure 2:**
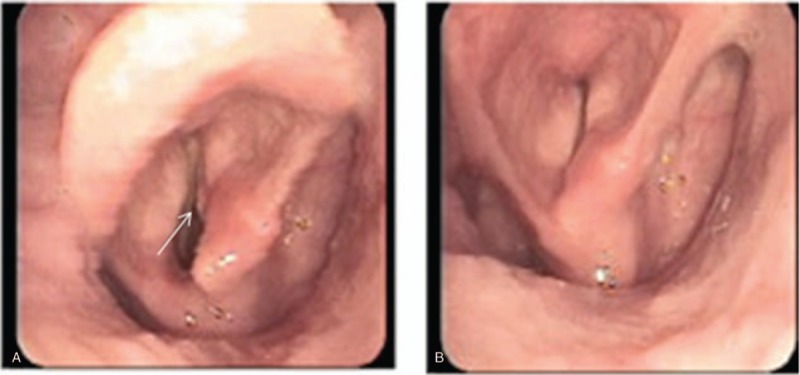
Electronic laryngoscope examination showed ventricular bands thickening, the ventricular bands compartment of the right side shows a rice-like projection, arytenoid region movement is poor. The arrow refers to the prominent tumor of the right ventricular band. A. Inspiration. B. Phonation.

**Figure 3:**
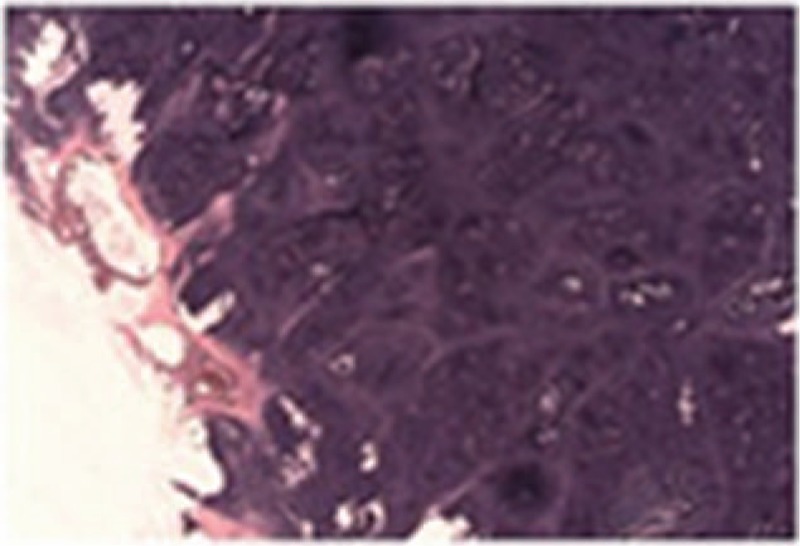
Postoperative pathology examination (hematoxylin and eosin stain, ×20) showed (right lobe of thyroid gland and larynx) chondroma.

A 73-year-old Chinese man was found a cervical mass for 1 month. He had a history of coronary heart disease for 2 years, but no relevant personal or family history of malignancy. Physical examination showed bilateral neck asymmetry, the trachea was deflected to the left, the carotid pulse was normal, approximately 6 × 5 cm solid masses were felt in the right lobe of the thyroid gland. The tumor mass border was unclear with smooth surface, and the tumor moved up and down with swallowing. The left lobe of the thyroid gland had no palpable mass and the neck had no palpable swollen lymph nodes. Accessory examination of neck color Doppler ultrasound at our hospital showed increase in size of the right lobe of the thyroid gland, which had abnormal shape. The upper right pole of the thyroid gland had an approximately 5.6 × 6.0 × 4.2 cm sized mass, with unclear margin, and multiple cystic nodules in the thyroid. The CT of thyroid showed increase in size of the right lobe of the thyroid gland, and an approximately 5.6 × 6.0 × 4.2 cm sized mass, the trachea was compressed and deflected to the left, the thyroid cartilage was compressed, deformed and reached the right subglottic region. The right thyroid lump had multiple calcifications, indicative of a tumor (Fig. 1). Electronic laryngoscope examination showed ventricular bands thickening, the right ventricular bands compartment showed a rice-like projection, arytenoid region movement was poor (Fig. 2). Thyroid function test was 5.07 mIU/L. The patient underwent subtotal thyroidectomy and excisional biopsy of neck mass under general anesthesia. Intraoperative findings indicated a hard mass in the thyroid gland area, the lateral border and the lower bound were closely related to the surrounding soft tissues, but there was an extremely close relationship between the medial border, the thyroid cartilage, and cricoid cartilage, with no gap between them. An ENT doctor was invited for intraoperative consultation, who suggested that some of the tumors should be removed and frozen. The rapid pathology of the tumor (thyroid right lateral lobe) indicated chondroma. The ENT doctor suggested laryngeal chondroma resection and tracheotomy under general anesthesia. Intraoperative findings indicated that the right lower half of the thyroid cartilage and the right half of the cricoid cartilage were chondroma, and complete resection of the tumor was performed. Postoperative pathology examination showed (right lobe of thyroid gland and larynx) chondroma (Fig. 3). Given the advanced age of the patient, long surgical duration and poor cardiorespiratory function, the patient suffered sudden cardiac death after the operation.

## 3 Discussion

Cartilaginous tumors of the larynx are rare; and only 250 cases have been reported since the original case presentation by Travers.^[2]^ They frequently occur in older patients, in the age range of 40 to 60 years, and the incidence is 3 times higher in males than in females. The etiology of laryngeal chondroma is unknown, and the most common etiology is irregular ossification of the laryngeal cartilage.^[1,3,4]^ Other etiologic factors are radiotherapy and teflon injection.^[5]^

Laryngeal chondroma constitutes only 0.12% of all head and neck tumors, and less than 1% of all laryngeal tumors.^[1,6,7]^ The tumor is mainly localized in posterior lamina of cricoid cartilage, followed by thyroid cartilage,^[5]^ epiglottis, and arytenoid cartilage.^[8,9]^ Laryngeal chondromas are usually slow growing, with variable clinical symptoms that are associated with tumor size and localization. If the tumor grows into the laryngeal cavity, its clinical manifestations include progressive hoarseness, dyspnea, and stridor. But, if it grows outside the larynx, it manifests as hard neck mass. Lewis et al^[10]^ reported that some laryngeal chondromas can also show dysphagia, about 15% of patients exhibit neck masses that move up and down with swallowing, which helps to differentiate them from a thyroid mass. Physical examination plays an important role in the diagnosis of laryngeal chondroma. Computed tomography (CT) scan is the most useful and reliable imaging method for laryngeal chondroma.^[1]^ Most chondromas appear as hypodense, expansive, and well-circumscribed masses on CT imaging, while speckled or patchy soft calcifications are seen in 75% to 80% of cases.^[11]^ In our case, the right thyroid lump with multiple calcifications suggested a tumor. Magnetic resonance imaging is infrequently used in laryngeal chondromas because of its insensitivity to chondroma calcification, but can aid in the diagnosis because of improved adjacent soft tissue resolution.^[12]^ However, pathological examination is necessary for the diagnosis of laryngeal chondroma. Its characteristic is that the tumor tissue is mainly composed of mature hyaline cartilage cells and cartilage matrix, the cartilage matrix is separated by sparse fibrous vascular tissue, the cartilage cells form cartilage tumor cell lacuna in the matrix. Laryngeal chondromas show a homogenous, monotonous pattern, with low cellularity (no more than 30–40 nuclei per high-power field), without pathological nuclear division. Low-power microscopy reveals a lobular growth pattern.^[1]^

The present case had an atypical history and laryngeal signs. The patient experienced neck mass only for 1 month, and presented with an active neck mass in the early stage. With disease progression, lump enlargement and growth into the laryngeal cavity was found. Color Doppler ultrasound showed parenchymal mass in the right thyroid gland. The CT of thyroid showed increase in the size of the right lobe of the thyroid gland. The thyroid cartilage was compressed, deformed and reached the right subglottic region. According to the size of the tumor and the anatomical center, the tumor may have originated from the thyroid gland. In the thyroid tumor, nodules and calcification are common, and most of them are benign. The thyroid gland is closely related to the thyroid cartilage, and the anatomic location of the tumor is near, the diagnostic source cannot be determined by radiographic examination, so it was initially misdiagnosed as right thyroid lobe tumor.

According to this case report and literature review, the diagnosis of the disease is based on the medical history, imaging, and pathological examination. Because this disease is rare, it is often misdiagnosed. Patients with hoarseness of voice due to unknown reasons, subglottic smooth mass, vocal cord paralysis, and cervical mass should be further examined by laryngofiberoscope. CT scan is also helpful for diagnosis of the disease. It can be used to determine the location, extent, calcification, and ossification of the tumor.

Conservation surgery, assuring the entire tumor resection including a clear margin of normal cartilage, is recommended treatment philosophy for laryngeal chondroma.^[5]^ Because laryngeal chondroma is benign, the principle of surgical treatment is to maximally remove the tumor, while preserving the laryngeal function. Laryngofissure or thyroid cartilage lateral incision are usually used to remove the tumor. Besides, total laryngectomy should be considered for laryngeal cartilage widespread violations and sarcomatoid tumors or recurrent laryngeal chondroma.

Laryngeal chondroma is extremely rare due to which it is easily neglected and misdiagnosed, especially in cases with atypical symptoms. Clinical presentation, laboratory examination, and CT scan are necessary for the diagnosis of laryngeal chondroma.

## Acknowledgments

The authors would like to thank our department colleagues and the devotion of this patient, and the patient has signed the informed consent form.

## Author contributions

**Data curation:** Zi Qiao Tan.

**Writing – original draft:** Zi Qiao Tan.

**Writing – review and editing:** Mengwei Yao, Tao Liu, Shaohua Wang, Jing Xu, Yonghong Zhang, Xinxin Yang, Dengdian Ma, Xiaoyu Li.
